# An efficient biological pathway layout algorithm combining grid-layout and spring embedder for complicated cellular location information

**DOI:** 10.1186/1471-2105-11-335

**Published:** 2010-06-18

**Authors:** Kaname Kojima, Masao Nagasaki, Satoru Miyano

**Affiliations:** 1 Human Genome Center, Institute of Medical Science, University of Tokyo, 4-6-1 Shirokanedai, Minato-ku, Tokyo 108-8639, Japan

## Abstract

**Background:**

Graph drawing is one of the important techniques for understanding biological regulations in a cell or among cells at the pathway level. Among many available layout algorithms, the spring embedder algorithm is widely used not only for pathway drawing but also for circuit placement and www visualization and so on because of the harmonized appearance of its results. For pathway drawing, location information is essential for its comprehension. However, complex shapes need to be taken into account when torus-shaped location information such as nuclear inner membrane, nuclear outer membrane, and plasma membrane is considered. Unfortunately, the spring embedder algorithm cannot easily handle such information. In addition, crossings between edges and nodes are usually not considered explicitly.

**Results:**

We proposed a new grid-layout algorithm based on the spring embedder algorithm that can handle location information and provide layouts with harmonized appearance. In grid-layout algorithms, the mapping of nodes to grid points that minimizes a cost function is searched. By imposing positional constraints on grid points, location information including complex shapes can be easily considered. Our layout algorithm includes the spring embedder cost as a component of the cost function. We further extend the layout algorithm to enable dynamic update of the positions and sizes of compartments at each step.

**Conclusions:**

The new spring embedder-based grid-layout algorithm and a spring embedder algorithm are applied to three biological pathways; endothelial cell model, Fas-induced apoptosis model, and *C. elegans *cell fate simulation model. From the positional constraints, all the results of our algorithm satisfy location information, and hence, more comprehensible layouts are obtained as compared to the spring embedder algorithm. From the comparison of the number of crossings, the results of the grid-layout-based algorithm tend to contain more crossings than those of the spring embedder algorithm due to the positional constraints. For a fair comparison, we also apply our proposed method without positional constraints. This comparison shows that these results contain less crossings than those of the spring embedder algorithm. We also compared layouts of the proposed algorithm with and without compartment update and verified that latter can reach better local optima.

## Background

For biological pathways such as signal transduction pathways, gene regulatory networks, and metabolic pathways, one of the crucial techniques for understanding their characteristics is to use graph visualization. Both publicly [1] and commercially available pathway databases [2] display retrieved pathways in the form of graphs to enable users to understand them easily. Usually, in these databases, a large number of pathways are retrieved with various types of criteria according to biologists' purposes. However, it is laborious to manually draw graphs for each request, and hence, automatic layout algorithms specialized for biological pathways are strongly desired.

Thus far, several types of drawing algorithms have been designed for biological pathways and they have been integrated in biological modeling and/or simulation software, e.g., Cell Illustrator [3,4], Pajek [5], PATIKA [6,7], and CADLIVE [8,9].

Karp and Paley extracted biological topologies such as linear, cyclic, and branching pathways and used them as the backbone of the layout [10]. For chemical reaction networks, Becker and Rojas proposed a method [11] that uses the longest directed cycle as the backbone of the layout to capture the flow of reactions. On the other hand, Wegner and Kummer used recursively extracted small cycles as the backbone of the layout [12], because such cycles are known to participate in important recycling processes. Their work has been implemented as an SBML application [13].

Several biological properties are considered in spring embedder approaches. The use of edge directions and simple positional constraints has been proposed for more general metabolic pathways [14,15]. In GOlorize [16], an additional attractive force is applied to nodes belonging to the same Gene Ontology class. An SBML layout extension, SBWAutoLayout [17], employs the spring embedder approach as its layout algorithm. Schreiber et al. [18] proposed to a generic layout algorithm where the spring force cost is optimized independently in horizontal and vertical directions. Due to the optimization strategy, the algorithm can handle placement constraints for the biological comprehension such as horizontal or vertical aligning of nodes, non-overlapping of nodes, and keeping the same network motifs to some formation. Spring embedder approaches are very popular in the field of Bioinformatics because of the harmonized appearance of their results. However, Li and Kurata noted that spring embedder approaches are not suitable for generating compact layouts of complex pathways [19]. In addition, such approaches have a difficulty in handling complicated positional constraints such as arranging some nodes only on a tours-shaped region, which corresponds to cellular membranes, e.g., nuclear inner membrane, nuclear outer membrane, and plasma membrane.

A grid layout algorithm for biological networks was first proposed by Li and Kurata, in which nodes of the given graph are mapped to grid points and the locally optimal mapping of nodes in terms of the defined cost function is searched over all possible mappings [19]. The cost function is defined by the weighted sum of several components: node distances weighted according to the graph structure and Manhattan distance [19], edge-edge and node-edge crossings [20], rewarding scores for the aligned nodes possessing the same biological attributes [21], and negative inner product between directions of in-edges and out-edges that induces the traceability of the flows [22]. Because finding optimal mapping is NP-hard [23] even when only edge-edge crossings are considered in the cost function, the basic grid layout algorithm repeatedly updates the layout by moving nodes one by one under a greedy search strategy, and a locally optimal layout is obtained after convergence. For efficient calculation, the cost differences calculated by checking movements of a node to a grid point at the current step are cached for calculating the cost differences at the next step [19]. Swapping the positions of two nodes is additionally considered at update steps for the better local optimum without increasing the time complexity [21], whereas Barsky et al. restricted movements of a node to stochastically selected grid points at update steps [24]. Further, the reduction of time complexity is accomplished by using sweep calculation algorithm [22], which can efficiently calculate edge-edge and node-edge crossings and the Manhattan distance. In addition, grid layout algorithms can deal with complicated positional constraints that are often assumed in biological networks as sub-cellular localization information, and thus, they succeed in generating compact and biologically comprehensible layouts.

We propose a new grid-layout-based spring embedder algorithm that considers the spring force cost as a component of the cost function of a grid-layout algorithm. Hence, the cost function consists of the spring force cost and edge-edge and node-edge crossings. As stated above, the sweep calculation can be used to efficiently count the number of edge-edge and node-edge crossings and calculate the Manhattan distance. However, the sweep calculation cannot be used to calculate the spring force. Therefore, we devise a new caching approach for calculating the spring force and propose a new layout algorithm having the same time complexity.

The remainder of this paper is organized as follows. In the Results and Discussion section, we discuss the performance of the proposed algorithm by comparing it with that of the conventional spring embedder approach on three biological networks. The conclusions of our work are presented in the Conclusions section. Finally, the Methods section describes the procedure of the proposed algorithm and its time complexity.

## Results and Discussion

### Experimental settings and results

We compare our proposed algorithm (Grid Layout) with a spring embedder layout algorithm [25] (Spring). As the attraction force *F*_*a*_(*d*) and repulsion force *F*_*r*_(*d*), *d*^2 ^and 1/*d*^2 ^are used, respectively, where *d *is the distance between nodes (here, the Euclidean distance is adopted). We use three biological networks that were constructed from curated knowledge in biological literatures:

• Endothelial cell model [26]: 221 nodes and 274 edges.

• Fas-induced apoptosis model [27]: 84 nodes and 93 edges.

• Cell fate simulation model of *C. elegans *[28]: 53 nodes and 59 edges.

Grid resolutions of 73 × 81, 39 × 29, and 26 × 21 are used for the endothelial cell model, Fas-induced apoptosis model, and cell fate simulation model of *C. elegans*, respectively. Both algorithms are implemented in Java and experiments were performed on a Core micro-architecture-based Xeon 3.0 GHz processor. For each model, ten random layouts are generated and applied to Grid Layout and Spring. Since node-edge crossings cause the difficulty on distinguishing the node connections, we consider node-edge crossings as more problematic factor than edge-edge crossings and then set more weight for node-edge crossings than edge-edge crossings in the cost function. Specifically, weight for node-edge crossings *w*_*n *_is two times as much as that for edge-edge crossing *w*_*e*_, i.e., *w*_*n *_= 2 × *w*_*e*_. For the three pathway networks, the numbers of rows and columns of the grid are determined by setting the grid interval as the size of basic elements and setting the canvas size as the size used in the manually created layout. Li and Kurata stated that the desirable numbers of rows and columns of grid are proportional to [19].

**Figure 1:**
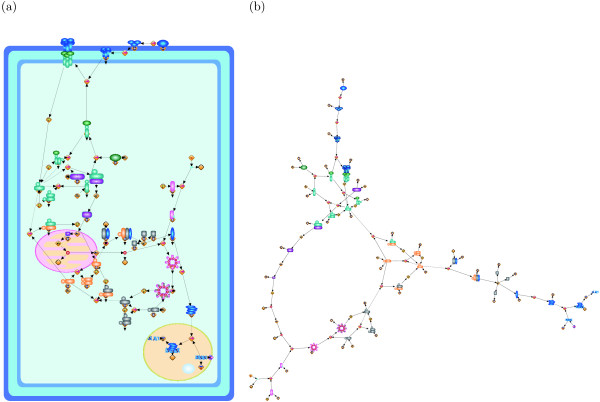
**Resulting layouts of Fas-induced apoptosis model obtained from Grid Layout (a) and Spring (b)**. Because the spring embedder algorithm does not consider location information, this location information is not shown in its resulting layout.

**Figure 2:**
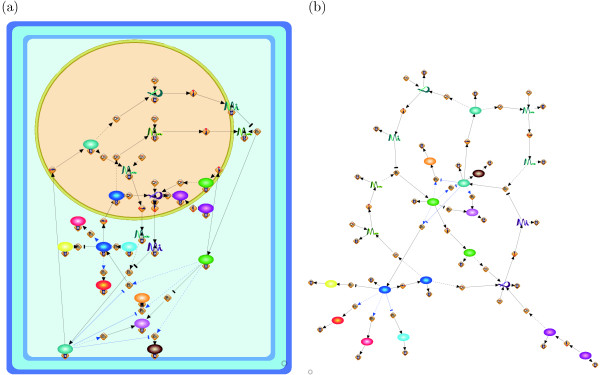
**Resulting layouts of cell fate simulation model of *C. elegans *obtained from Grid Layout (a) and Spring (b)**. Because the spring embedder algorithm does not consider location information, this location information is not shown in its resulting layout.

**Figure 3:**
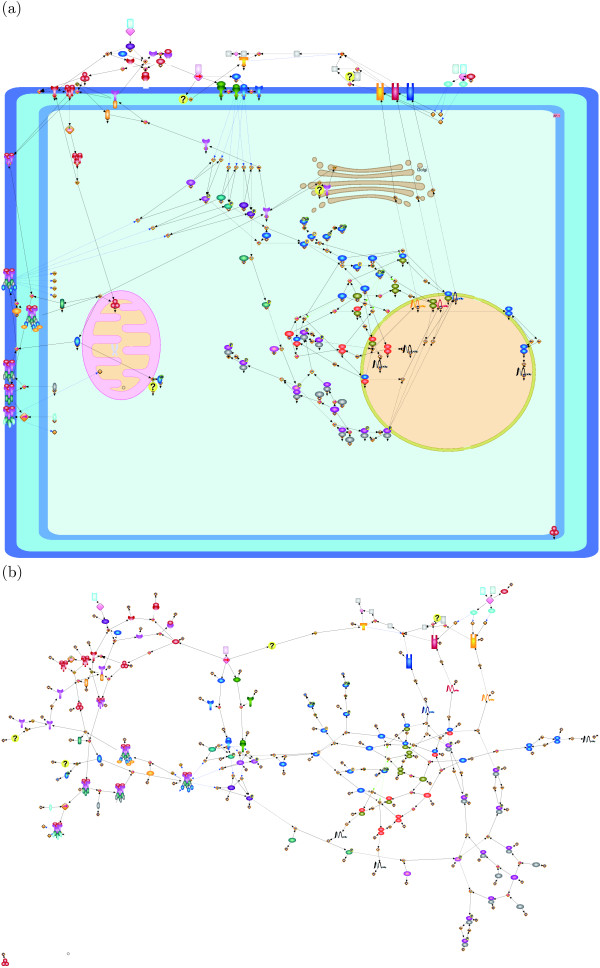
**Resulting layouts of endothelial cell model obtained from Grid Layout (a) and Spring (b)**. Because the spring embedder algorithm does not consider location information, this location information is not shown in its resulting layout.

Since by calculating (the number of row + the number of columns)  for the three pathways we have (73 + 81)/ = 10.76, (39 + 39)/ = 7.42, (26 + 21)/ = 6.46, our grid resolutions somehow follow the assumption in [19]. The repulsion force is defined to be inversely proportional to the square of distance between nodes, and the force comes from all nodes. As we discussed, the grid size is proportional to  and the repulsion force does not depend on the grid size if nodes are evenly distributed in the canvas ((1/)^2 ^× |*V*| = 1). Thus, we use the same weight for repulsion force (*w*_*r *_= 1) among three networks. Remnant weights to be adjusted are attraction force *w*_*a *_and edge-edge crossings *w*_*e*_. We empirically select their parameter ranges as *w*_*a *_= {1, 5, 10, 12} and *w*_*e *_= {10, 50}, respectively. Figures 1, 2, and 3 respectively show the layouts of the Fas-induced apoptosis model, cell fate simulation model of *C. elegans*, and endothelial cell model obtained from Grid Layout and Spring, which has the minimum cost among the results from ten random layouts. Note that here only a resulting layout under one of the above parameter sets is given for each model (for Fas-induced apoptosis model *w*_*a *_= 1, *w*_*r *_= 1, *w*_*e *_= 10, *w*_*n *_= 20; cell fate simulation model of *C. elegans w_a _*= 1, *w*_*r *_= 1, *w*_*e *_= 50, *w*_*n *_= 100; and endothelial cell model *w*_*a *_= 12, *w*_*r *_= 1, *w*_*e *_= 50, *w*_*n *_= 100). For results under other parameter sets, see Section 1 of Additional file 1.

**Figure 4:**
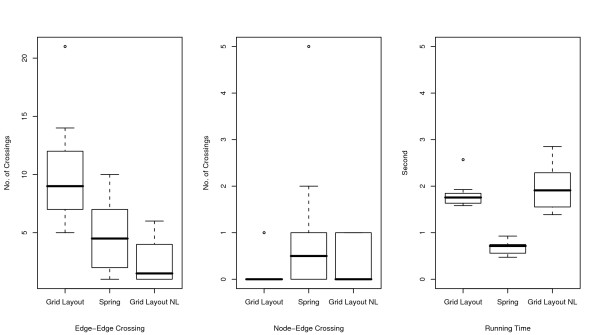
**Comparisons of number of edge-edge crossings (left), number of node-edge crossings (middle), and running time (right) for Fas-induced apoptosis model**. Numbers of edge-edge intersections and node-edge intersections and running time for Grid Layout, Spring, and Grid Layout NL (Grid Layout considering no location information) are compared using box plots. These indicators are obtained by applying these three algorithms to ten randomly obtained layouts of the Fas-induced apoptosis model.

**Figure 5:**
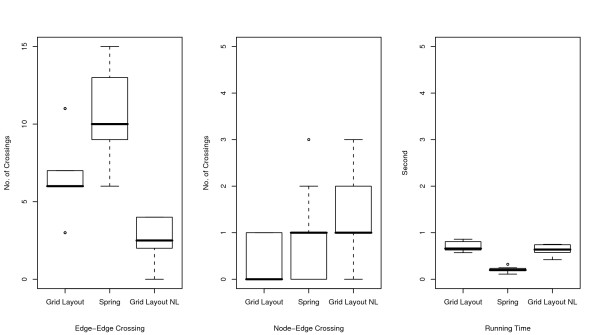
**Comparisons of number of edge-edge crossings (left), number of node-edge crossings (middle), and running time (right) for cell fate simulation model of *C. elegans***. Numbers of edge-edge intersections and node-edge intersections and running time for Grid Layout, Spring, and Grid Layout NL (Grid Layout considering no location information) are compared using box plots. These indicators are obtained by applying these three algorithms to ten randomly obtained layouts of the cell fate simulation model *C. elegans*.

**Figure 6:**
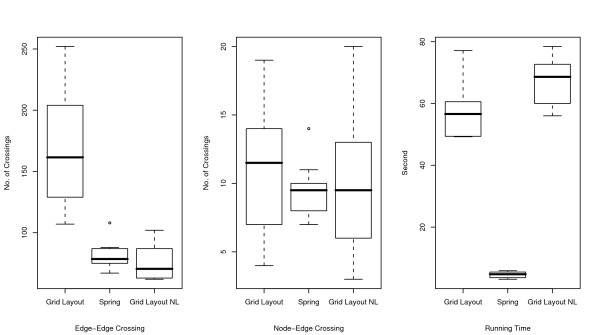
**Comparisons of number of edge-edge crossings (left), number of node-edge crossings (middle), and running time (right) for endothelial cell model**. Numbers of edge-edge intersections and node-edge intersections and running time for Grid Layout, Spring, and Grid Layout NL (Grid Layout considering no location information) are compared using box plots. These indicators are obtained by applying these three algorithms to ten randomly obtained layouts of the endothelial cell model.

The resulting layouts are generated using an XML format called Cell System Markup Language (CSML), and these can be directly displayed by using the Cell Illustrator Player in a Web browser. All URL links are listed in Table 1. In the layouts, the cellular membrane, nucleus, mitochondria, and Golgi apparatus are depicted by a blue frame, yellow circle, red oval, and brown crab-shaped object, respectively. In order to analyze the number of crossings in the layouts and the running time, ten random layouts for each model are also applied to Grid Layout without positional constraints (hereafter called Grid Layout NL). For these random layouts, we compare the numbers of edge-edge and node-edge crossings in the resulting layouts and the running time of Grid Layout and Spring. These comparisons are summarized in Figures 4, 5, and 6 for the Fas-induced apoptosis model, cell fate simulation model of *C. elegans*, and endothelial cell model, respectively.

**Table 1:** Summary of layout results.

Model/Algorithm	URL
Fas-induced apoptosis model/Grid Layout	https://cionline.hgc.jp/cifileserver/launchCIOPlayer?mode=BP&antialias=on&model=https://www.csml.org/download/model/csml30/gl/apoptosisgrid.csml

Fas-induced apoptosis model/Spring	https://cionline.hgc.jp/cifileserver/launchCIOPlayer?mode=BP&antialias=on&model=https://www.csml.org/download/model/csml30/gl/apoptosisspring.csml

Fas-induced apoptosis model/GDC	https://cionline.hgc.jp/cifileserver/launchCIOPlayer?mode=BP&antialias=on&model=https://www.csml.org/download/model/csml30/gl/apoptosisgdc.csml

cell fate simulation model of *C. elegans*/Grid Layout	https://cionline.hgc.jp/cifileserver/launchCIOPlayer?mode=BP&antialias=on&model=https://www.csml.org/download/model/csml30/gl/elegansgrid.csml

cell fate simulation model of *C. elegans*/Spring	https://cionline.hgc.jp/cifileserver/launchCIOPlayer?mode=BP&antialias=on&model=https://www.csml.org/download/model/csml30/gl/elegansspring.csml

cell fate simulation model of *C. elegans*/GDC	https://cionline.hgc.jp/cifileserver/launchCIOPlayer?mode=BP&antialias=on&model=https://www.csml.org/download/model/csml30/gl/elegansgdc.csml

endothelial cell model/Grid Layout	https://cionline.hgc.jp/cifileserver/launchCIOPlayer?mode=BP&antialias=on&model=https://www.csml.org/download/model/csml30/gl/endothelialgrid.csml

endothelial cell model/Spring	https://cionline.hgc.jp/cifileserver/launchCIOPlayer?mode=BP&antialias=on&model=https://www.csml.org/download/model/csml30/gl/endothelialspring.csml

endothelial cell model/GDC	https://cionline.hgc.jp/cifileserver/launchCIOPlayer?mode=BP&antialias=on&model=https://www.csml.org/download/model/csml30/gl/endothelialgdc.csml

The resulting layouts of Grid Layout and Spring for the Fas-induced apoptosis model, cell fate simulation model of *C. elegans*, and endothelial cell model are available at the following links.

As shown in the two left-hand side plots of Figures 4, 5, and 6, the resulting layouts from Grid Layout tend to contain more edge-edge and node-edge crossings than those from Spring. This may be because positional constraints restrict the search space of Grid Layout. This hypothesis is also reinforced by the results of Grid Layout NL, which contains a lesser or, occasionally a comparable number of crossings as compared to those of the spring embedder algorithm among three cases on both edge-edge and node-edge crossings.

Although the above comparison appears to suggest that positional constraints degrade the quality of the resulting layouts, in the next subsection, we show that how location information serves to improve the understandability of biological networks while surveying the results of Grid Layout.

### Dynamic resizing and repositioning of compartments

**Figure 7:**
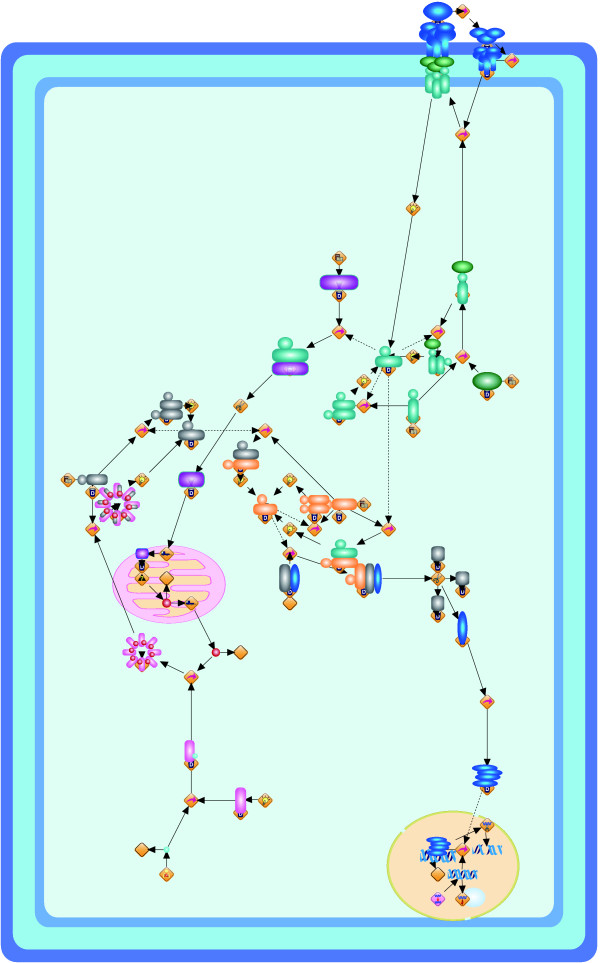
**Resulting layouts of Fas-induced apoptosis model obtained from GDC (Grid Layout with dynamic compartment update)**. Iterative update of the sizes and positions of nucleus and mitochondria is considered at each step.

**Figure 8:**
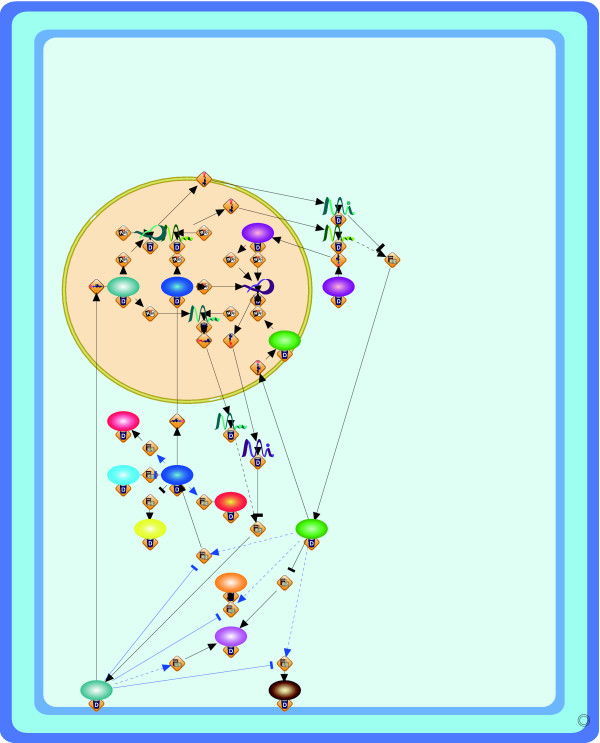
**Resulting layouts of cell fate simulation model of *C. elegans *obtained from GDC (Grid Layout with dynamic compartment update)**. Iterative update of the size and position of nucleus is considered at each step.

**Figure 9:**
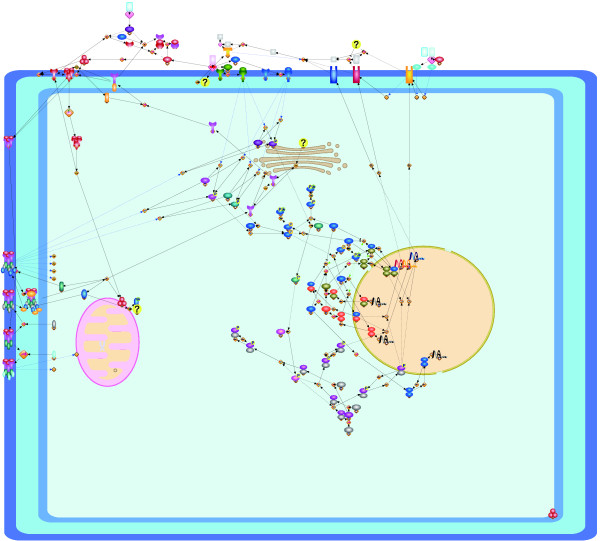
**Resulting layouts of endothelial cell model obtained from GDC (Grid Layout with dynamic compartment update)**. Iterative update of the sizes and positions of nucleus, mitochondria, and Gologi apparatus are considered at each step.

**Figure 10:**
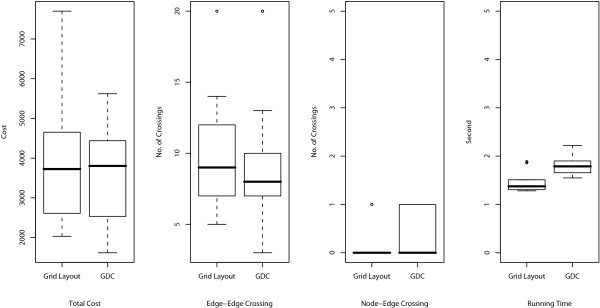
**Comparisons of total cost (left), number of edge-edge crossings (middle left), number of node-edge crossings (middle right), and running time (right) for Fas-induced apoptosis model**. Total costs, Numbers of edge-edge intersections and node-edge intersections and running time for Grid Layout and GDC (Grid Layout with dynamic compartment update) are compared using box plots. These indicators are obtained by applying these two algorithms to ten randomly obtained layouts of the Fas-induced apoptosis model.

**Figure 11:**
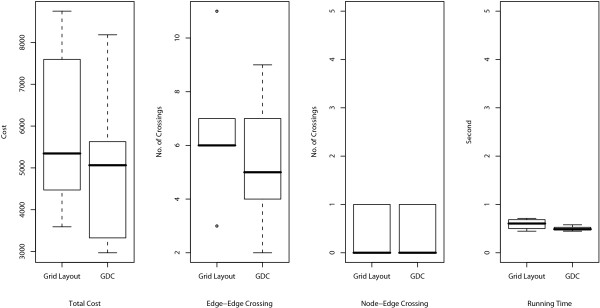
**Comparisons of total cost (left), number of edge-edge crossings (middle left), number of node-edge crossings (middle right), and running time (right) for cell fate simulation model of *C. elegans***. Total costs, Numbers of edge-edge intersections and node-edge intersections and running time for Grid Layout and GDC (Grid Layout with dynamic compartment update) are compared using box plots. These indicators are obtained by applying these two algorithms to ten randomly obtained layouts of the cell fate simulation model *C. elegans*.

**Figure 12:**
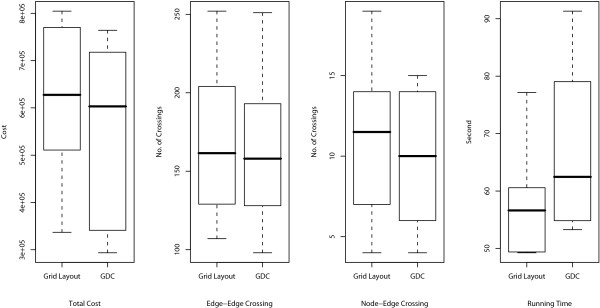
**Comparisons of total cost (left), number of edge-edge crossings (middle left), number of node-edge crossings (middle right), and running time (right) for endothelial cell model**. Total costs, Numbers of edge-edge intersections and node-edge intersections and running time for Grid Layout and GDC (Grid Layout with dynamic compartment update) are compared using box plots. These indicators are obtained by applying these two algorithms to ten randomly obtained layouts of the endothelial cell model.

Dynamic resizing and repositioning of compartments are considered in our proposed algorithm. Li and Kurata [19] stated that as an empirical rule, setting vertical and horizontal sizes of canvas proportional to the square root of the number of nodes is suitable for most networks. This rule can also be applied to size the compartment according to the nodes localized in it. However, if nodes in a compartment are densely connected, they tend to create cluster, and thus they do not fill out the space optimally. By making the size of the compartment smaller, better quality layout will be obtained. On the other hand, if nodes are distributed uniformly enough in the compartment, its enlargement might be required for the better quality of the layout. Also, if the center of the compartment is away from the nodes' center of gravity, it might spoil the quality of the resulting layout. Thus, we consider dynamic update of sizes and positions of compartments iteratively at each step. Hereafter, we call Grid Layout with dynamic compartment update as GDC. Figures 7, 8, and 9 show the minimum cost resulting layouts obtained from GDC for Fas-induced apoptosis model, cell fate simulation model of *C. elegans*, and endothelial cell model, respectively. Weights for the cost functions are the same as those of the experiments in the previous section. Initial sizes and positions of the compartments are the same as in layouts for Grid Layout. In this study, we keep the size and position of the extracellular or cellular membrane, and then update the sizes and positions of other components inside of the cellular membrane such as nucleus, mitochondria, and Golgi apparatus. The detailed procedures of dynamic compartment update are in the following method section. As a common property in the layouts of the three models, nodes of the layouts of GDC are centered on each compartment, whereas nodes of the layouts Grid Layout tend to be positioned only on a part of each compartment. In addition, the compartments are well resized and then are filled out with the nodes enough, e.g., nodes on nucleus in Figure 9, comparing to nodes on nucleus in the layout image of Figure 3(a). We also compare the total cost, the number of edge-edge crossings, the number of node-edge crossings, and computational time of the resulting layouts of Grid Layout and GDC. The box plots of total cost, the number of edge-edge crossings, the number of node-edge crossings, and computational time are summarized in Figures 10, 11, and 12. Although GDC requires slightly more computational time than Grid Layout, GDC provides better or competitive results in other indicators. Since the repositioning of compartments is allowed in GDC, the positions of compartments are moved to more desirable positions, which contributes to the better cost, the number of edge-edge crossings, and the number of node-edge crossings. On the other hand, since the consideration of the dynamic compartment update spreads out the search space of the layouts, the more steps are required to reach local optima.

## Discussion

The first model shown in Figure 1 is a famous signal transduction pathway, apoptosis, which is known to participate in various biological processes such as development, maintenance of tissue homeostasis, and elimination of cancer cells. Malfunctions of apoptosis have been implicated in many forms of human diseases such as neurodegenerative diseases, AIDS, and ischemic stroke. Apoptosis is reportedly caused by various inducers such as chemical compounds, proteins, or removal of NGF. The biochemical pathways of apoptosis are complex and depend on both the cells and the inducers. In particular Fas-induced apoptosis has been studied in detail and its simulation model has been proposed [27]. Fas ligands, which usually exist as trimmers in the extracellular region, bind and activate their receptors by inducing receptor trimerization in the cytoplasm membrane region. Activated receptors recruit adaptor molecules such as Fas-associating protein with death domain (FADD), which recruit procaspase-8 to the receptor complex, where it undergoes autocatalytic activation in the cytoplasm. Activated caspase-8 activates caspase-3 through two pathways. In the complex pathway, caspase-8 cleaves the Bcl-2 interacting protein and its COOH-terminal part translocates to the mitochondria where it triggers the release of cytochrome c. The cytochrome c released from the mitochondria binds to apoptotic protease activating factor-1 (Apaf-1) together with dATP and procaspase-9 and activates caspase-9 in the cytoplasm. Caspase-9 cleaves procaspase-3 and activates caspase-3. In other pathway, caspase-8 cleaves procaspase-3 directly and activates it. In the nucleus, caspase-3 cleaves DNA fragmentation factor (DFF) 45 in a heterodimeric factor of DFF40 and DFF45. The cleaved DFF45 dissociates from DFF40, inducing the oligomerization of DFF40 that has DNase activity. The active DFF40 oligomer causes internucleosomal DNA fragmentation, which is an apoptotic hallmark indicative of chromatin condensation. As stated above, these reaction events are strictly regulated in specific cellular locations, and therefore, the corresponding location information cannot be ignored in the resulting layout. Figure 1(a) clearly shows the regulation of these events in each cellular location, i.e., plasma membrane, cytoplasm, mitochondria, and nucleus. In contrast, Figure 1(b) shows the two different flows by Fas-induced apoptosis; however, it is difficult to capture the location information of each event.

Figure 2 shows the cell fate determination model of two gustatory neurons of *C. elegans *- ASE left (ASEL) and ASE right (ASER) [28]. These neurons are morphologically bilaterally symmetric but physically asymmetric in function, and their fates are strictly regulated by the double negative feedback loop (DNFL), the main path of which consists of four steps: (i) activation of DIE-1 protein leads to the activation of *lsy-6 *miRNA in the nucleus; (ii) *lsy-6 *miRNA is transported from the nucleus and inhibits the translation of *cog-1 *mRNA into COG-1 protein; (iii) if the COG-1 protein is not suppressed, then it is translocated into the nucleus and activates the transcription of *mir-273 *miRNA in the nucleus; and (iv) *mir-273 *miRNA is transported from the nucleus and inhibits the translation of *die-1 *mRNA; this completes the loop to (i). In a manner similar to apoptosis, these DNFL reaction events are strictly regulated in specific cellular locations, and therefore, the corresponding location information cannot be ignored in the resulting layout. Although Figure 2(b) shows these steps, it is difficult to capture the location information of each step. For instance, most of the proteins and miRNAs in this model, e.g., COG-1 protein, LIM-6 protein, DIE-1 protein, *mir-273 *miRNA, and *lsy-6 *miRNA, translocate between the nucleus and the cytoplasm. However, such information cannot be inferred from this figure. In contrast, Figure 2(a) shows the regulations of these steps while keeping the cellular location of each step, i.e., cytoplasm and nucleus.

These differences can be observed more clearly in larger models. Figure 3 shows the responses of endothelial cells to the tumor necrosis factor, with an emphasis on the induction of endothelial leukocyte adhesion molecules with more elements than in the other two models [26]. Since adhesion molecules are usually localized on the plasma membrane, many molecules should be on the plasma membrane domain. Usually, external signals are received by these adhesion molecules and transferred into the molecules located in the cytoplasm. Finally, these signals trigger the translation of mRNAs in the nucleus. From the viewpoint of the density of nodes, Figure 3(b) appears to suitably keep a uniform density of nodes. Unfortunately, in terms of the understanding of cascading events, Figure 3(b) does not provide completely useful information because, due to the lack of location information, it is difficult to interpret the network as the response model by the tumor necrosis factor from the external region to the nucleus via the cytoplasm. On the other hand, as shown in Figure 3(a), our layout requires no difficulty in tracing the flow of biological cascading events in our layout, i.e., a reader can easily interpret the network as the response model of a cell from the external region to the nucleus via the cytoplasm as a signal flow.

## Conclusions

We propose a grid-layout-based spring embedder algorithm that exploits the advantages on both methods, i.e., consideration of location information and harmonized layouts. Not only the harmonized appearance of resulting layouts, spring force also contributes the reduction of crossings, which is verified by comparing two cases of grid layout algorithms: (i) without considering distance cost and (ii) considering only spring force. (Section 2 of Additional file 1). Although only spring force is considered as the distance cost in this study, we can incorporate other distance cost such as the Manhattan distance cost in the cost function simultaneously.

In addition, to explicitly consider the reduction of crossings, edge-edge and node-edge crossing costs are included in the cost function. To calculate spring forces among nodes, we proposed an efficient calculation method for spring force cost with *O*(|*V*|^2^·*h*·*w*), and to calculate other costs, we employ the sweep calculation [22], which can count the crossings for all the possible movements of a node at once. By applying the proposed algorithm and spring embedder algorithm to three biological networks, we verified that the consideration of location information significantly improves the understandability of a network from a biological viewpoint.

In order to realize better biological pathway layouts, under the framework of grid layout, several useful cost functions were proposed, e.g., (i) rewarding score for aligned nodes in one line with the same attribute and (ii) negative inner product of directions of in-edge and out-edges. Feature (i) is very important for biologists because the nodes in a biological pathway usually have biological attributes, e.g., a node is mRNA, protein, modified protein, or complex of proteins, and they explicitly distinguish these components. Feature (ii) is also very important for biochemists because it helps in understanding the reaction flows of the biological pathway. These cost functions can be easily plugged in to our grid layout algorithm without increasing the time complexity. Furthermore, to obtain a better resulting layout, we can also introduce the swapping operation of nodes at each step of moving a node to a vacant grid point to increase the search space while keeping the time order.

Our proposed algorithm succeeded in realizing the required features for biological pathway layouts; however, several enhancements are still required. For example, usually, the combination and order of some biological reactions can be grouped, and thus, this set of reactions, e.g., phosphorylation and dephosphorylation, and related biological elements, e.g., protein and modified protein, can be considered as subgraphs that consume several grid points. Although in our search strategy the final result might fall into bad local optima, for the better local optimum, we can use simulated annealing or other techniques which enables escape from the bad local optima although more computational time is required for the final result. If we could extend the current grid layout algorithm to allow the movement of multiple fixed structured nodes at once, then the required feature would be realized. Our layout framework assumes that compartments representing sub-cellular localizations are allocated by users in advance and then the layout algorithm is applied, but we also considered dynamic adjustment of sizes and positions of these compartments. In this work, the initial state of compartments are given in advance. For automatically providing their initial state, the following approach can be considered as an example. The size of compartment can be determined by the square root of the number of nodes that localize in the same compartment. For the positions of the compartments, we put pair of compartments in close positions if many edges are bridging them.

In addition, the bending of edges that enables bypassing edge-edge and node-edge crossings has not been considered in the current grid layout algorithms. This could be achieved by considering bends as virtual nodes and handling them in a manner similar to normal nodes in search steps.

## Methods

Given a graph *G *= (*V, E*) and a grid of *h *rows and *w *columns, we define a cost function for mappings of nodes to grid points and show an algorithm that finds the mapping of nodes, minimizing the cost function in a greedy manner. The cost function is defined by the weighted sum of four components:

**(a) ***Attraction force F_a_*(*d*(*P *(*v*), *P *(*u*))) between pairs of adjacent nodes *v *and *u *in the graph *G*, where *P *(*v*) and *P *(*u*) are grid points to which *v *and *u *are mapped, respectively, and *d*(*P *(*v*), *P *(*u*)) is the distance between two grid points *P *(*v*) and *P *(*u*).

**(b) ***Repulsion force F_r_*(*d*(*P *(*v*), *P *(*u*))) between any pairs of nodes *v *and *u*.

**(c) ***Number of edge-edge crossings *∑_*e.f*∈*E*_*I*_*e*_(*e*, *f*), where *I*_*e*_(*e, f *) is a binary function that returns 1 if *e *and *f *cross with each other and 0 otherwise.

**(d) ***Number of node-edge crossings *∑_*u*∈*V*,*e*∈*E*_*I*_*n*_(*v*, *e*) where *I*_*n*_(*v*, *e*) is a binary function that returns 1 if *v *and *e *cross with each other and 0 otherwise.

Formally, the cost function is given by(1)

where (*v*) is the set of adjacent nodes of *v*, and *w*_*a*_, *w*_*r*_, *w*_*e*_, and *w*_*n *_∈ *R*^+ ^are weights for the components.

### Search algorithm

In grid layout, nodes are mapped to different grid points, i.e., no grid point is occupied by more than one node. Our algorithm optimizes the cost function by moving a node to an empty grid point at each step in a greedy manner. Note that, given positional constraints, nodes are allowed to be moved only to empty grid points satisfying the positional constraints, e.g., if a node is localized only in the cellular membrane, it can be mapped only to those grid points corresponding to cellular membrane. The above operation can be performed by calculating *delta cost*, which is the cost difference by the movement of a node to a grid point, for all nodes and for all vacant grid points. Although a naïve algorithm requires *O*(|*V*|^2^·*h*·*w*) time to find the movement that reduces the cost most at each step, we devise an efficient method that requires *O*(|*E*|^2^·min(*h*, *w*) + *h*·*w*) time for finding the movement, which is described below.

### Efficient calculation of spring force

Repulsion force for a node *v *is given by

where the function *P *(*i*) returns the grid point to which *i *∈ is mapped. Checking the movement of a node to all the vacant grid points requires |*V*|·*h*·*w *calculations, and hence, *O*(|*V*|^2^·*h*·*w*) time is required in total at each step.

Although the above naïve calculation has a higher time complexity than existing grid layout algorithms, we propose an efficient calculation. When *v *is moved from *P *(*v*) to *q*, the repulsion force for *v *is given by:

Because the term ∑_*u*∈*V*_*F*_*r*_(*d*(*q*, *P*(*u*))) in the above equation depends on *q*, but not on *v*, by calculating ∑_*u*∈*V*_*F*_*r*_(*d*(*q*, *P*(*u*))) for all the vacant points *q *initially, the calculation of *c*_*r*_(*v*) requires a constant time. The term ∑_*u*∈*V*_*F*_*r*_(*d*(*q*, *P*(*u*))) for all the vacant points requires *O*(|*V*|·*h*·*w*) time, and |*V*|·*h*·*w *movements are considered at each step. Therefore, in total, *O*(|*V*|·*h*·*w*) time is required at each step to calculate the repulsion force.

For the attraction force, the delta cost Δ_*v*, *p *_induced by the movement of a node *v *to grid point *p *can be calculated by considering the attraction force between *v *and its adjacent nodes. In addition, the movement of a node *v *influences the delta costs only for *v *and its adjacent nodes, i.e., the delta costs for its non-adjacent nodes at the previous and current steps are the same. Thus, by using the cached delta costs obtained at the previous step, we can calculate the delta costs efficiently. If *v *is moved from *p *to *q *at the previous step, the delta cost for the movement of *v *to *r *can be updated by

and for a node *u *in (*v*) to *r*,

### Efficient counting of edge-edge and node-edge crossings

The delta cost caching technique is used for counting crossings as well. When *v *is moved at the previous step, the following cases need to be considered for calculating the delta costs induced by the movement of node *u*.

**(i) **edge-edge crossing between *e*_*u *_∈ *E*_*u *_and *e*_*v *_∈ *E*_*v*_, where *E*_*v *_and *E*_*u *_are the sets of edges connected to *v *and *u*, respectively.

**(ii) **node-edge crossing between *e*_*u *_∈ *E*_*u *_and *v*.

**(iii) **node-edge crossing between *e*_*v *_∈ *E*_*v *_and *u*.

**(iv) **edge-edge crossing between edge *e*(*u*, *v*) and *E*\(*E*_*u *_∪ *E*_*v*_) if edge *e*(*v*, *u*) exists.

**(v) **node-edge crossing between edge *e*(*u*, *v*) and *V*\{*v*, *u*} if edge *e*(*v*, *u*) exists.

In a naïve way, the crossings of the above cases are counted in each movement of a node to a grid point. Thus, the above cases (i), (ii), (iii), (iv), and (v) may respectively require *O*(|*E*_*u*_||*E*_*v*_|), *O*(|*E*_*u*_|), (|*E*_*v*_|), *O*(*E*), and *O*(|*V*|) time. Thus, each movement of a node *u *requires *O*(|*E*_*u*_||*E*_*v*_|) time if *u *∈ (*v*) and *O*(|*E*_*u*_||*E*_*v*_| + |*E*|) time otherwise. Hence, in total, *O*(*h*·*w*·deg(*v*)|*E*|) time is required at each step, where deg(*v*) is the degree of *v*.

These time complexities can be reduced by using more sophisticated crossing counting algorithms [29-31]. In this study, we employ the sweep calculation algorithm [22], which is known to require less time complexity than even sophisticated crossing counting algorithms under the assumption that *h *and *w *are proportional to  and the average degree is bounded by *O*(|*V*^1/4^). The grid resolution in the former assumption is commonly employed in existing grid layout algorithms [19-22]. In addition, because the biological networks we are motivated to tackle can be modeled as scale-free networks whose average degree is bounded by a constant value [32], the latter assumption is reasonable.

Given an edge *e*, a node *v *connected with *e*, and a set of edges *F *⊆ *E *on the grid, we consider the counting of crossings between *e *and edges in *F *for the movement of *v *to each grid point. Unlike conventional crossing counting algorithms, the sweep calculation can simultaneously count the crossings for all the movements of *v *in *O*(|*F*|·min(*h*, *w*) + *h*·*w*) time [22]. Because node-edge crossings can be counted in a manner similar to the case of edge-edge crossings, by replacing the number of edges with the number of nodes, the time complexity for counting node-edge crossings is obtained. Therefore, for the five cases mentioned above, the sweep calculation simultaneously counts crossings for mappings of *u *to *q *for all grid points *q *in *O*(|*E*_*u*_||*E*_*v*_|·min(*h*, *w*) + *h*·*w*), *O*(|*E*_*u*_|·min(*h*, *w*) + *h*·*w*), *O*(|*E*_*u*_|·min(*h*, *w*) + *h*·*w*), *O*(|*E*|·min(*h*, *w*) + *h*·*w*), and for (v) *O*(|*V*|·min(*h*, *w*) + *h*·*w*) time, respectively. Thus, the algorithm using sweep calculation requires *O*(deg(*v*)|*E*|·min(*h*, *w*) + *h*·*w*·|*V*|) time at each step.

### Time complexity at the initial step

The calculation of delta costs at the initial step requires more computational time than those at latter steps because no cached delta costs are available. Here, the time complexity for the first step is analyzed for each component.

**(a) Repulsion force**: The computation of repulsion forces does not rely on the cached delta costs. Thus, *O*(|*V*|·*h*·*w*) time is required.

**(b) Attraction force**: Because attraction forces between a node *v *and its adjacent nodes (*v*) are calculated, *O*(deg(*v*)) time is required for each movement of *v*. Thus, *O*(|*E*|·*h*·*w*) time is required in total.

**(c) Edge-edge crossing**: Because crossings between edges in *E*_*v *_and other edges are checked for the movement of a node *v*, *O*(|*E*|^2^·min(*h*, *w*) + *h*·*w*) time is required by sweep calculation.

**(d) Node-edge crossing**: When a node *v *is moved, we need to consider two cases: (i) crossings between edges in *E*_*v *_and all nodes other than *v*, and (ii) crossings between *v*, and all the edges other than edges in *E*_*v*_. Thus, *O*(|*E*||*V*|·min(*h*, *w*) + *h*·*w*) time is required by sweep calculation.

From the above analysis, the proposed algorithm requires *O*(|*E*|^2^·min(*h*, *w*) + *h*·*w*) time at the initial step.

### Procedures for resizing and repositioning of compartments

The resizing and repositioning of compartments are mainly comprised of the following procedures:

**(i) **The size of each compartment is updated according to the distribution range of nodes localized in the compartment.

**(ii) **The position of the compartment is updated in such a way that the center of the compartment is close to the center of gravity of nodes localized to it.

For the resizing of each compartment in step (i), we fist calculate  and  where *v*_*c *_is a node localized to the compartment *c*, *b*_*c *_is the center of gravity of *v*_*c *_(*d *the nodes localized to *c*, and *d*_*v*_(·,·) and *d*_*h*_(·,·) return vertical and horizontal distance of *v*_*c *_and *b*_*c*_, respectively. Then, if *s*_*v *_< 0.4 × **the width of the compartment **and *s*_*h *_< 0.4 × **the height of the compartment**, the compartment is shrunk to one level smaller size (0.95 times as large as the current size, in our setting). On the other hand, *s*_*v *_< 0.9 × the width of the compartment and 2 value *s*_*h *_< 0.9 × the height of the compartment, the compartment is enlarged to one level larger size (1/0.95 time as large as the current size). For the limitation of the scaling, the compartment cannot be shrunk if its current size is smaller than 0.6 times of its original size, while it cannot be enlarged if its size is larger than 1.5 times of its original size.

For step (ii), the position of the compartment that minimizes the distance of the center of compartment and the center of gravity of nodes are searched. For an easier implementation, we discredited the center of compartment and the center of gravity of nodes to some grid points and employed the Manhattan distance for the distance measure. Positioning is searched in the limited distance from the center of gravity, which is set to 10 in our setting. if the compartment is resized. Also, for the search procedure, the following two conditions must be satisfied:

• Every node satisfies its localization information.

• No compartments are allow to overlap.

For the efficiency and simplicity of checking the second condition, we only consider overlapping of the rectangles that surround the compartments. Overlapping of these rectangles can be detected by checking if at one of four corners are in the other rectangle. If no valid position can be found in the above procedure, the size of the compartment is turned back to its previous size of step (i) and then step (ii) is applied again. If no valid position is still not found, then its current size and position are used for the next step. When several nodes are located close to the surface of a compartment, its size and position cannot be updated to a better condition as resizing and repositioning of the compartment violate the localization of these nodes. In order to avoid the case, we introduce the following cost function to nodes located within one grid distance from the surface of the compartments defined as *α*·exp(-*βl*), where *α *and *β *are respectively set to 20·(*w *+ *r*) and 0.002 from an empirical rule and *l *is the number of updated steps. Due to the above cost function, the placement of nodes close to the surface of the compartments is avoided and then the compartments can be updated to a better size and position with higher probability. In addition, since the above cost function converges to zero with increasing update steps *l*, the convergence of the search is guaranteed.

Next, we consider the time complexity of the dynamic compartment update. For step (i), the calculation of *s*_*h *_and *s*_*c *_require *O*(|*V*_*c*_|) time for a compartment *c*, where *V*_*c *_is the set of nodes localized to *c*. Resizing the compartment *c *requires *O*(*w*_*c*_·*h*_*c*_) time, where *w*_*c *_and *h*_*c *_are width and height of the compartment *c*. Thus, in total, *O*(|*V*| + *w*·*h*) = *O*(*w*·*h*) time is required for step (i). For step (ii), checking the violation of localization information of every node requires *O*(|*V*) time for each movement of a compartment even in a naïve way. In addition, at worst, each compartment is moved to all the grid points in the limited distance from the center of gravity and the number of them are obviously less than the number of grid points. Checking the overlapping of a pair of compartment requires constant time. Since the number of compartments are limited (in our setting, at most three), which can be considered as a constant, the time complexity of step (ii) requires *O*(*w*·*h*·|*V*|) time at worst case. Actually, since the number of grid points searched for the repositioning of compartments are limited, the time complexity for the dynamic compartment update is not heavy in practice, which is supported by the comparison of running time of the proposed algorithm with and without the dynamic compartment update in Figure 10, 11, and 12.

## Authors' contributions

KK and MN discussed the main direction of this work. SM gave idea to this work for the further improvement. The main engine of search algorithm was implemented by KK and the visualization engine was implemented by MN as a Cell Illustrator plug-in. The final manuscript was read and approved by all authors.

## Supplementary Material

Additional file 1 **Comparison of the resulting layouts under several parameter sets (Section 1) and among three cost functions (Section 2)**. Layouts of Fas-induced apoptosis model, cell fate simulation model of *C. elegans*, and endothelial cell model obtained by the proposed algorithm under several parameter sets are compared in Section 1. From the comparison, the influence of parameters to positions of nodes and the number of crossings are discussed. In Section 2, resulting layouts of Grid Layout, Grid Layout without considering spring force cost, and Grid Layout considering spring force cost are compared on the three models. By using box plots for the numbers of edge-edge and node-edge crossings on layouts from these algorithms, the effectiveness of spring force cost is discussed. Click here for file
