# Supplementary material for: An efficient biological pathway layout algorithm combining grid-layout and spring embedder for complicated cellular location information
Source: BMC Bioinformatics. 2010 Jun 18;11:335. doi: 10.1186/1471-2105-11-335 (PMC2904761; doi:10.1186/1471-2105-11-335)
Supplement: Additional file 1 — Comparison of the resulting layouts under several parameter sets (Section 1) and among three cost functions (Section 2). Layouts of Fas-induced apoptosis model, cell fate simulation model of C. elegans, and endothelial cell model obtained by the proposed algorithm under several parameter sets are compared in Section 1. From the comparison, the influence of parameters to positions of nodes and the number of crossings are discussed. In Section 2, resulting layouts of Grid Layout, Grid Layout without considering spring force cost, and Grid Layout considering spring force cost are compared on the three models. By using box plots for the numbers of edge-edge and node-edge crossings on layouts from these algorithms, the effectiveness of spring force cost is discussed. [file 1471-2105-11-335-S1.PDF]

Supplemental Material for An Efficient Biological Pathway Layout Algorithm Combining Grid-layout and Spring Embedder for Complicated Cellular Location Information

Kaname Kojima, Masao Nagasaki, Satoru Miyano

1 Discussion for the resulting layouts under several parameter sets

We apply our proposed algorithm to three biological networks, endothelial cell model, Fas-induced apoptosis model, and cell fate simulation model of *C. elegans* with the following parameters: $w_r = 1$, $w_a = 0.1, 1, 5, 12$, $w_e = 10, 50$, and $w_n = 2 \cdot w_e$. For the details of the three models and parameters, see the main manuscript. For each parameter set, we select the layout of the minimum cost among results obtained from ten randomly generated initial layouts. Figures 1, 2, and 3 show the best layout under parameter set for endothelial cell, Fas-induced apoptosis model, cell fate simulation model of *C. elegans*, respectively. As there are eight combinations for these parameter set, eight layouts are provided for each model, i.e, each figure. As a commonly observed property in the three models, nodes are densely positioned for the higher attraction force, while nodes are sparsely positioned for the lower attraction force. To analyze the difference of the number of crossings by the crossing weights w_e and w_n, we also summarize the number of crossings under eight parameter sets for each model in Tables 1, 2, and 3. From these tables, we can conclude that the resulting layouts under higher crossing weight tend to contain less crossings on both edge-edge and node-edge cases than those under lower crossing weight, which is especially observed in the results of endothelial cell model. However, for Fas-induced apoptosis model, results under higher crossing weight contain more crossings than those under less crossing weight in some cases. This is probably due to the fact that the number of crossings are reduced enough even under the lower crossing weight and search algorithm in the higher crossing weight cases accidently fall into bad local optima.

Table 1: The numbers of edge-edge crossings and node-edge crossings in the resulting layouts of endothelial cell model under several parameter sets.

parameter set	# of edge-edge crossings	# of node-edge crossings
$w_a = 0.1, w_e = 10$	133	3
$w_a = 1, w_e = 10$	128	7
$w_a = 5, w_e = 10$	190	24
$w_a = 12, w_e = 10$	201	28
$w_a = 0.1, w_e = 50$	111	2
$w_a = 1, w_e = 50$	131	1
$w_a = 5, w_e = 50$	105	5
$w_a = 12, w_e = 50$	129	9

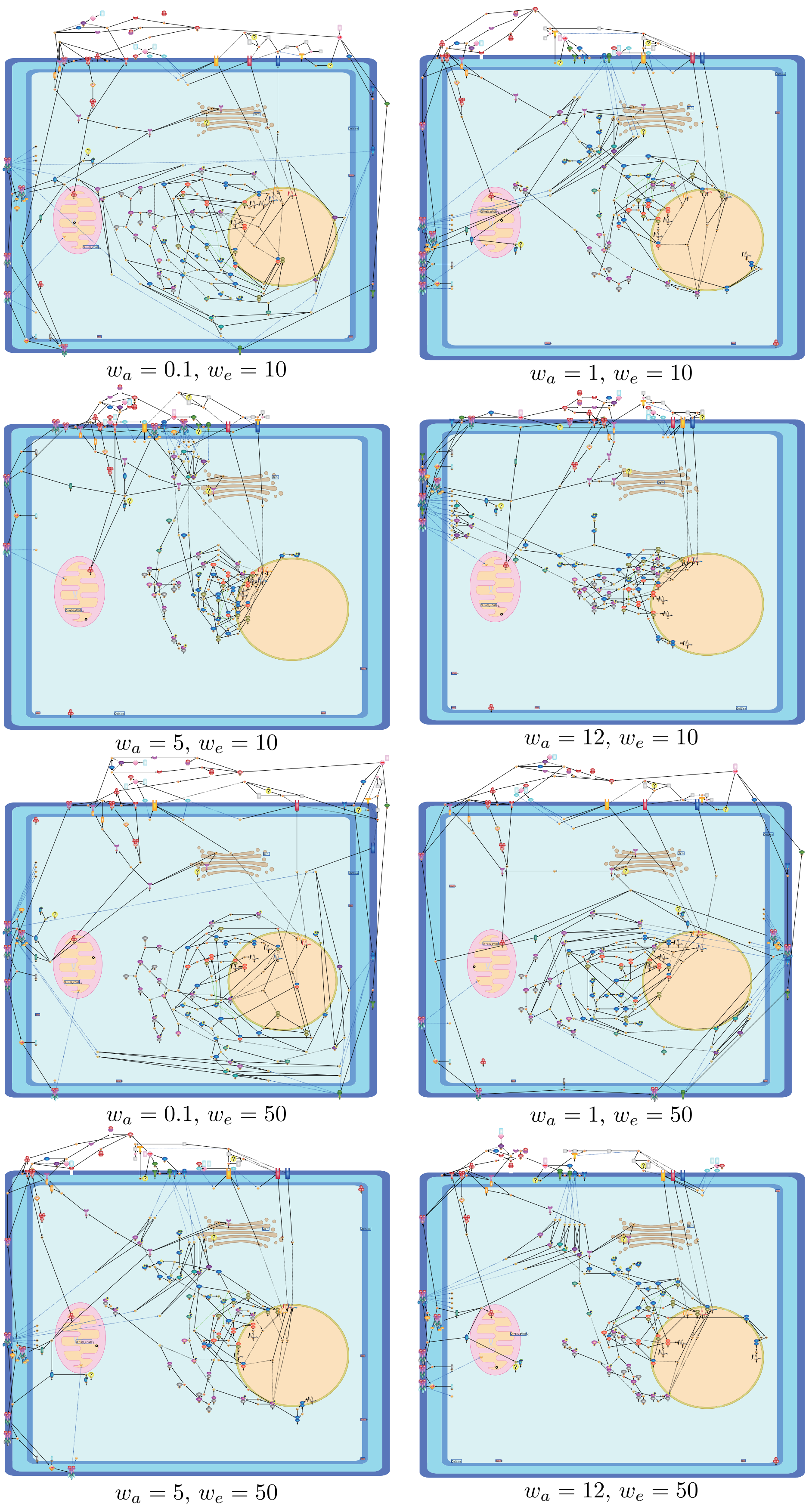

Figure 1: Eight resulting layouts for endothelial cell model. The applied parameter set is given below the layout image.

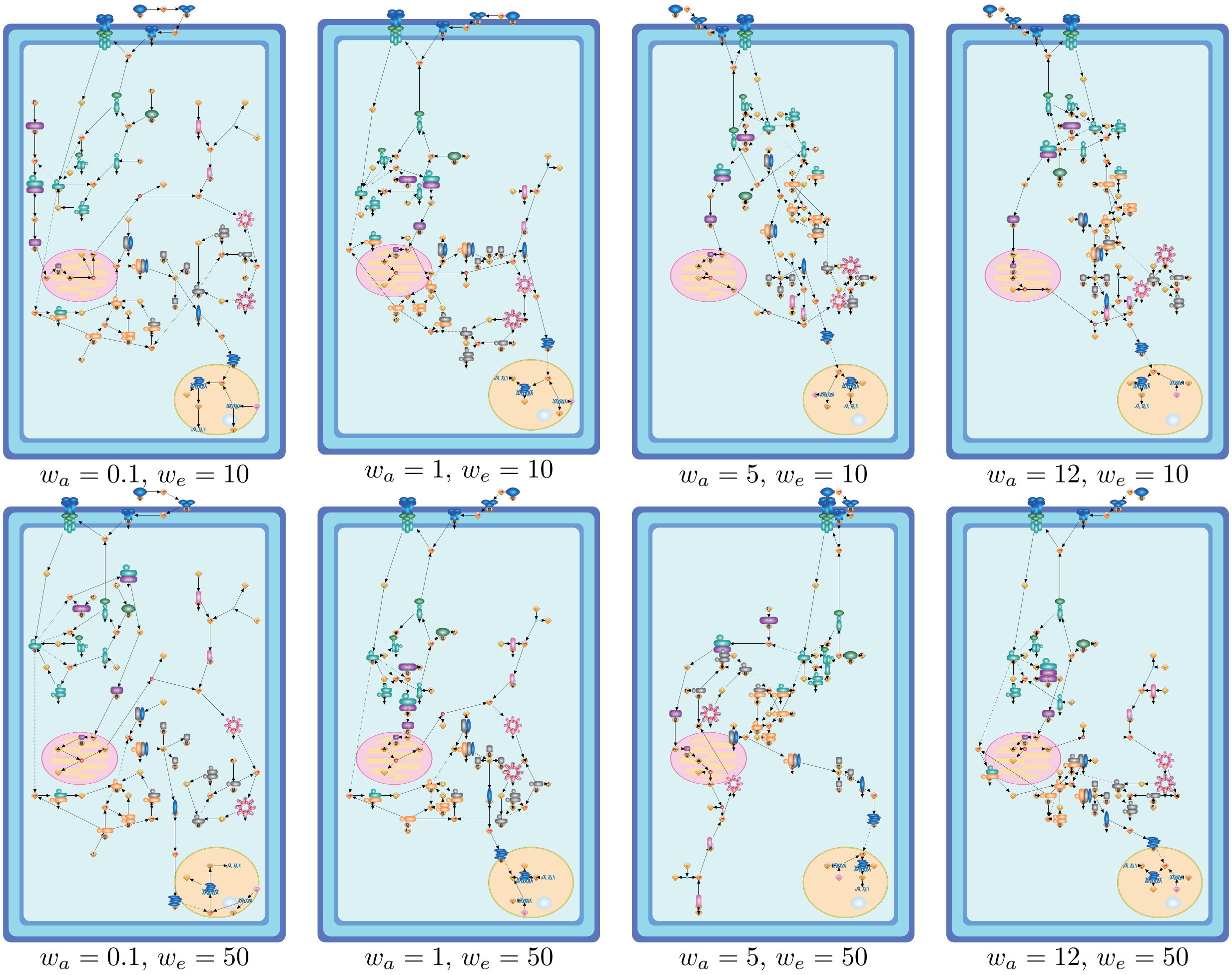

Figure 2: Eight resulting layouts for Fas-induced apoptosis model. The applied parameter set is given below the layout image.

Table 2: The numbers of edge-edge crossings and node-edge crossings in the resulting layouts of Fas-induced apoptosis model under several parameter sets.

parameter set	# of edge-edge crossings	# of node-edge crossings
$w_a = 0.1, w_e = 10$	7	0
$w_a = 1, w_e = 10$	9	0
$w_a = 5, w_e = 10$	8	0
$w_a = 12, w_e = 10$	22	3
$w_a = 0.1, w_e = 50$	7	0
$w_a = 1, w_e = 50$	12	0
$w_a = 5, w_e = 50$	10	0
$w_a = 12, w_e = 50$	16	0

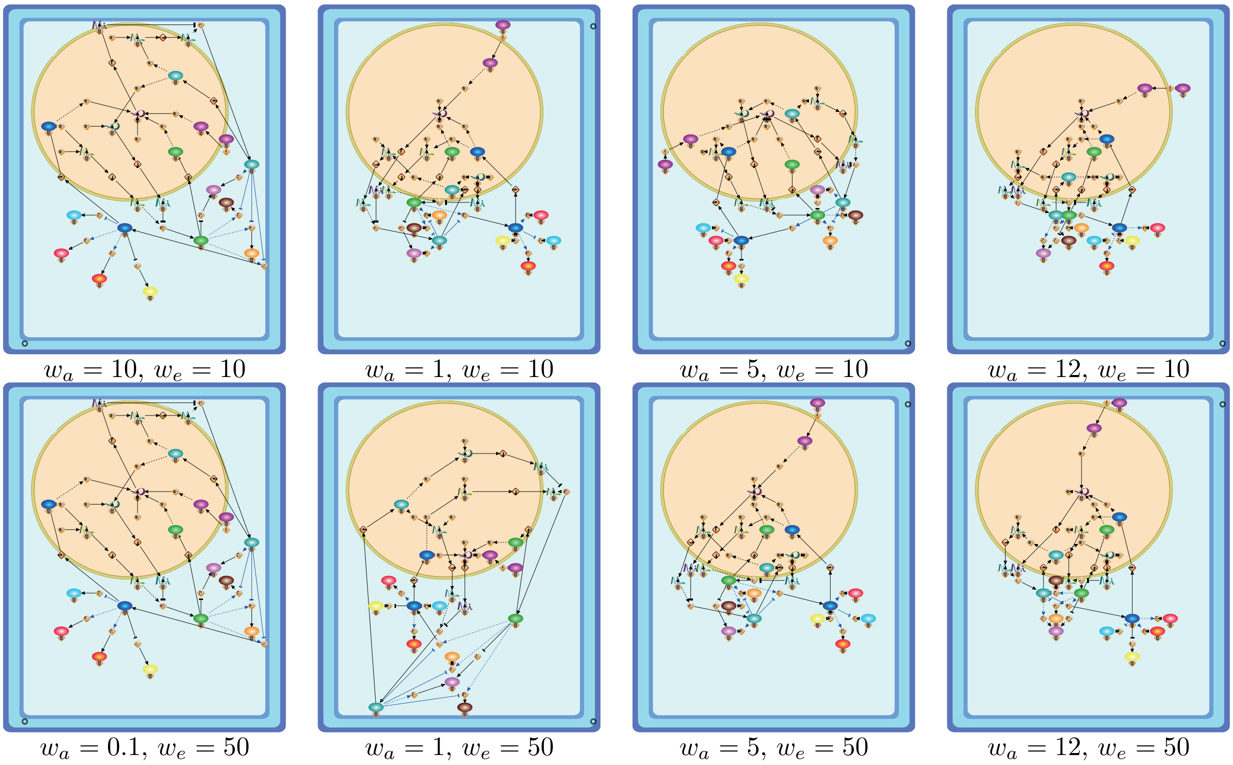

Figure 3: Eight resulting layouts for cell fate simulation model of *C. elegans*. The applied parameter set is given below the layout image.

Table 3: The numbers of edge-edge crossings and node-edge crossings in the resulting layouts of Cell fate simulation model of *C. elegans* under several parameter sets.

parameter set	# of edge-edge crossings	# of node-edge crossings
$w_a = 0.1, w_e = 10$	15	0
$w_a = 1, w_e = 10$	14	0
$w_a = 5, w_e = 10$	17	3
$w_a = 12, w_e = 10$	20	4
$w_a = 0.1, w_e = 50$	15	0
$w_a = 1, w_e = 50$	11	1
$w_a = 5, w_e = 50$	14	0
$w_a = 12, w_e = 50$	14	1

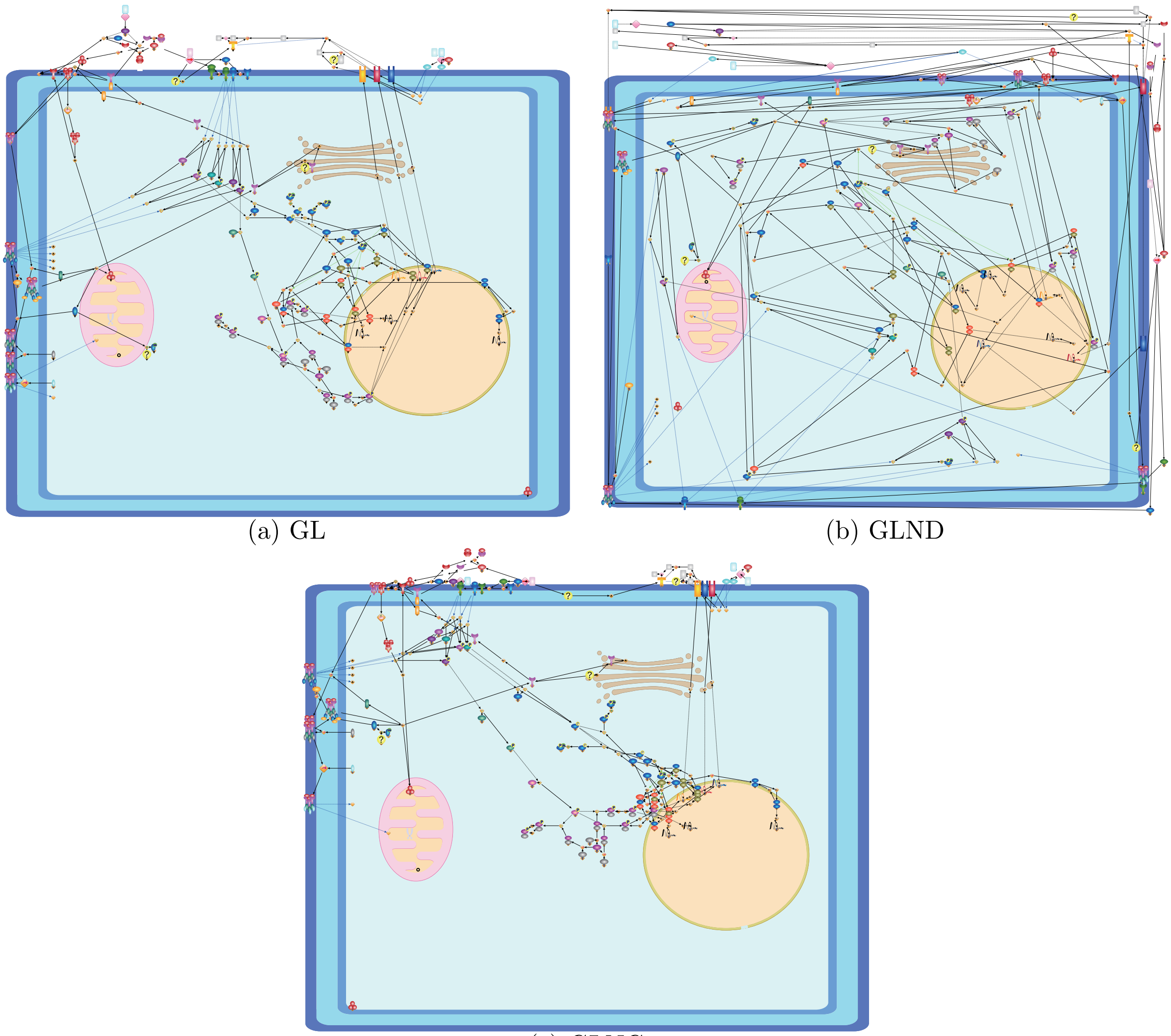

(a) GL (b) GLND

(c) GLNC

Figure 4: Comparison of resulting layouts of three cases for endothelial cell model. (a) A resulting layout of GL. (b) A resulting layout of GLND. (c) A resulting layout of GLNC.

2 Discussion for the effectiveness of spring force

In order to verify the effectiveness of spring force, we apply the proposed algorithm (GL), without considering distance cost (GLND), and considering only sprint force (GLNC) to the three models and compare their resulting layouts. For GL, weight parameters for the cost functions are the same as those in the main manuscript. Crossing cost weights w_e and w_n for GLND are the same as GL, but w_a and w_r are set to zero, while for GLNC, w_e and w_n are set to zero and w_a and w_r are the same as those of GL. The resulting layouts of the minimum cost from these algorithms for endothelial cell model, Fas-induced apoptosis model, and cell fate simulation model of *C. elegans* are summarized in Figures 4, 5, and 6, respectively. The numbers of edge-edge and node-edge crossings of layouts from GL, GLND, and GLNC on these models are also summarized with box plots in Figures 7(a), (b), and (c), respectively. The resulting layouts of GLND are very messy comparing to layouts from the other two algorithms. In addition, in Fas-induced apoptosis model and cell fate simulation model of *C. elegans*, the layouts of GLND contain more edge-edge crossings than those of GLNC, which does not consider the cost for crossings. The resulting layouts of GLNC have the harmonized and simple appearance due to the effect of spring force and their understandabilities seem high. However, the layouts of GLNC tend to contain more crossings on both between edges and between edge and nodes than those of GL. This fact implies that actual traceability of edges are low. On the other hand, the resulting layouts of GLNC are simpler and more compact comparing to those of the other two algorithms. The layouts of GL contain minimum number of edge-edge crossings in all

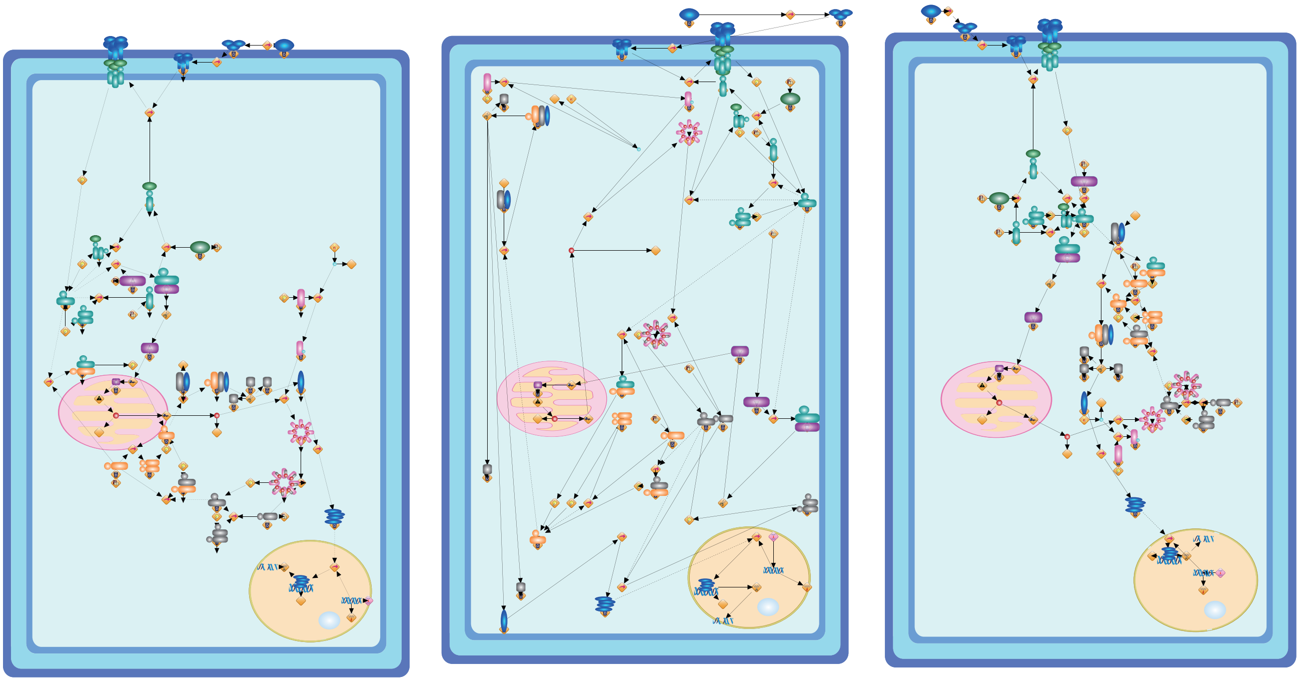

(a) GL (b) GLND (c) GLNC

Figure 5: Comparison of resulting layouts of three algorithms for Fas-induced apoptosis model. (a) A resulting layout of GL. (b) A resulting layout of GLND. (c) A resulting layout of GLNC.

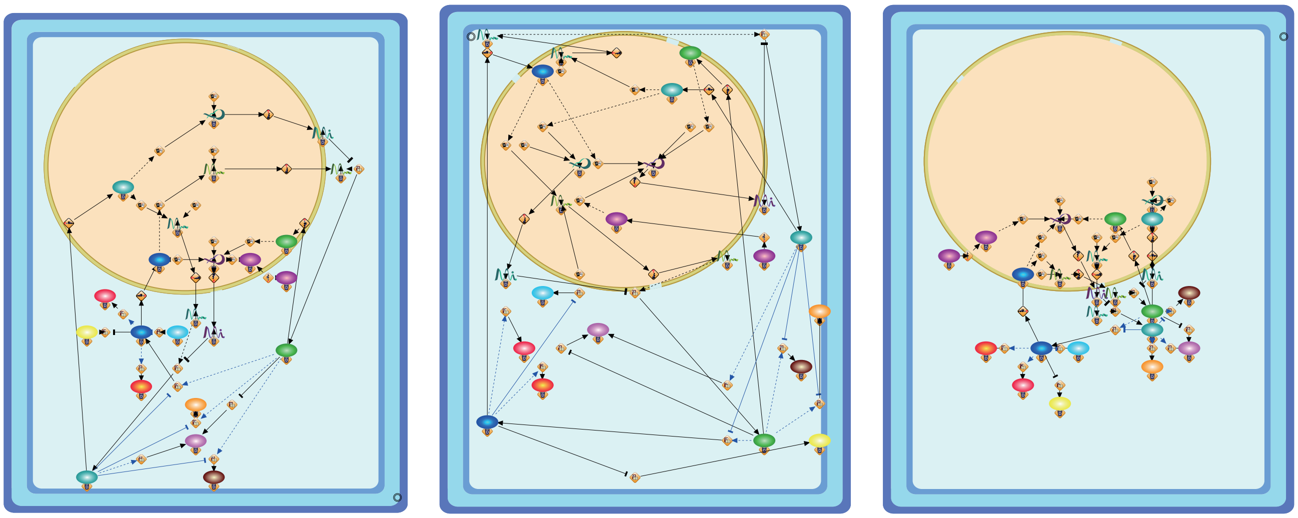

(a) GL (b) GLND (c) GLNC

Figure 6: Comparison of resulting layouts of three algorithms for cell fate simulation model of *C. elegans*. (a) A resulting layout of GL. (b) A resulting layout of GLND. (c) A resulting layout of GLNC.

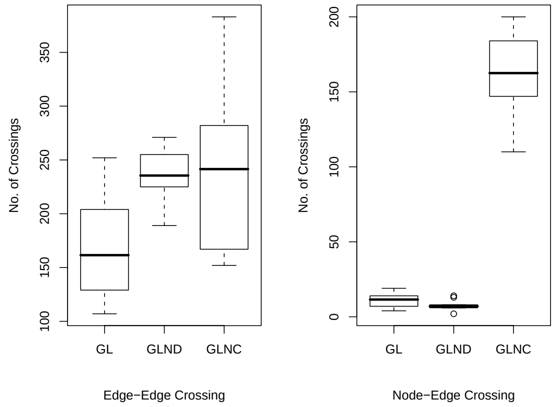

(a) Endothelial cell model

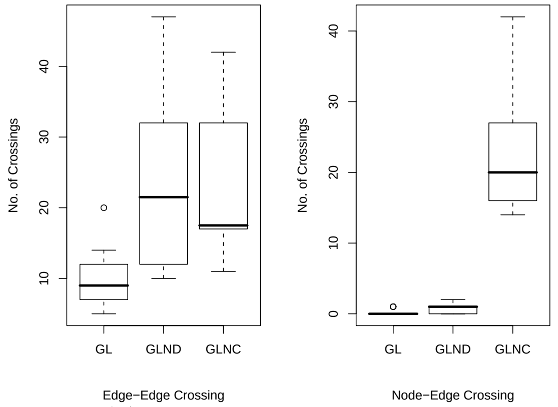

(b) Fas-induced apoptosis model

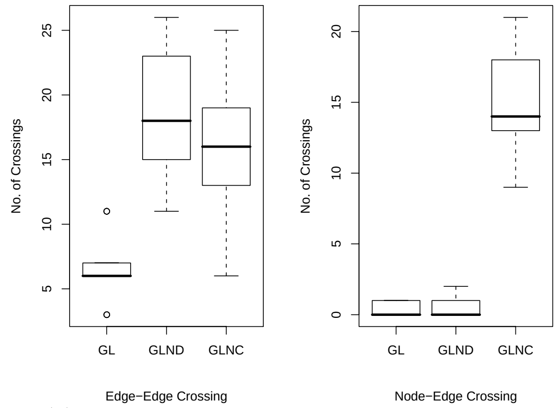

(c) Cell fate simulation model of *C. elegans*

Figure 7: Comparison of number of edge-edge crossings (left) and number of node-edge crossings (right) for GL, GLND, and GLNC on (a) endothelial cell model, (b) Fas-induced apoptosis model, and (c) cell fate simulation model of *C. elegans*. These indicators are obtained by applying these three algorithms to ten randomly obtained layouts on each model.

models and for node-edge crossings, GL is better than GLND on cell fate simulation model of *C. elegans* and is competitive with GLND on the other two models. Thus, from the comparison, we concluded that spring force contributes the reduction of crossings and it is important to consider both spring force and crossings in the cost function.
